# Biomarkers and Mental Disorders: A Relevance Analysis Using a Random Forest Algorithm

**DOI:** 10.3390/biom15060793

**Published:** 2025-05-29

**Authors:** Joice M. A. Rodolpho, Krissia F. Godoy, Bruna D. L. Fragelli, Jaqueline Bianchi, Fernanda O. Duarte, Luciana Camillo, Gustavo B. Silva, Paulo H. M. Andrade, Juliana A. Prado, Carlos Speglich, Fernanda F. Anibal

**Affiliations:** 1 Laboratory of Inflammation and Infectious Diseases, Federal University of São Carlos (UFSCar), São Carlos 13565-905, SP, Brazil; 2 Center for Development of Functional Materials, Federal University of São Carlos (UFSCar), São Carlos 13565-905, SP, Brazil; 3 Polytechnic School-Data Science and Artificial Intelligence, Pontifical Catholic University of Campinas (PUC Campinas), Campinas 13087-571, SP, Brazil; 4 Department of Medicine, Federal University of São Carlos (UFSCar), São Carlos 13565-905, SP, Brazil; 5 Leopoldo Américo Miguez de Mello Research Center CENPES/Petrobrás, Rio de Janeiro 21941-915, RJ, Brazil

**Keywords:** depression, anxiety, cytokines, IL-6, TNF, vitamin D, cortisol, biomarkers, machine learning, random forest

## Abstract

Depression and anxiety are mental health disorders that significantly impact global public health, affecting more than 280 million people with depression and 301 million with anxiety worldwide. These conditions impair individuals’ ability to engage in economic and personal activities and can lead to severe outcomes, such as suicide. Current research suggests that inflammatory cytokines, such as interleukin-1β (IL-1β), interleukin-6 (IL-6), and tumor necrosis factor (TNF), play crucial roles in the pathophysiology of these disorders, influencing neurotransmitters. Elevated cortisol levels, typically associated with anxiety, worsen these conditions through dysregulation of the hypothalamic–pituitary–adrenal (HPA) axis. Additionally, vitamin D deficiency has been linked to reduced production of dopamine and norepinephrine, hormones involved in depressive symptoms. This study utilized the Random Forest machine learning algorithm along with cross-validation to assess the importance of various biomarkers, including IL-1β, IL-6, IL-8, TNF, cortisol, vitamin D, NT-proBNP, CK-MB, troponin, myoglobin, and C-reactive protein (CRP), in volunteers of both sexes diagnosed with mental disorders. A single sample from each of the 96 participants was analyzed, consisting of 50 women and 46 men. The results revealed sex-specific differences in biomarker relevance, with vitamin D, CRP, and D-dimer being the most predictive for depression in men, while IL-6, CRP, and vitamin D were significant in women. For anxiety, vitamin D and myoglobin were important biomarkers in men, while IL-8 and vitamin D were key in women. The methodological strategy adopted, based on the use of Random Forest and cross-validation assessment, not only confirmed the robustness of the model but also reliably identified the most important biomarkers for the outcomes studied.

## 1. Introduction

Depression and anxiety are among the most prevalent psychiatric disorders and are associated with a high psychosocial burden and a significant reduction in the quality of life of affected individuals [1,2]. It is estimated that more than 280 million people worldwide live with depression, while approximately 301 million suffer from anxiety disorders [3]. Projections indicate that by 2030, depression may become the leading cause of global disability [4].

This scenario becomes even more concerning regarding the current economic instability and the lasting effects of the COVID-19 pandemic. The signs and symptoms of these disorders compromise not only individual functionality but also generate significant social and economic impacts, and in more severe cases, may lead to suicide [5]. Studies show that up to 60% of individuals suffering from depression also exhibit characteristic symptoms of anxiety [6,7,8].

In depression, individuals often experience a persistently low mood or apathy, along with changes in appetite, weight, sleep, and energy levels, as well as cognitive symptoms such as mental slowing, low self-esteem, hopelessness, and suicidal behavior [9,10]. Anxiety, on the other hand, involves excessive apprehension, fear, and worry—whether generalized or specific—accompanied by somatic symptoms such as dizziness, shortness of breath, chest pain, and palpitations [11].

Despite the distinction between the two conditions, symptoms often overlap and lack specificity, making diagnosis challenging and compromising both treatment planning and prognosis [12]. This symptomatic similarity makes clinical management more complex, especially in mental health settings with multiple vulnerabilities [13]. The presence of psychiatric and medical comorbidities further exacerbates the condition, reinforcing the need for an integrated and multidisciplinary approach [14].

In this context, it is strategic to develop means of refining diagnosis as well as quantitatively monitoring clinical progression and treatment response, situations in which biomarkers are promising.

Inflammatory cytokines have been widely discussed in depression and anxiety because they act on neurotransmitters, further exacerbating the effects of these conditions [15].

The levels of interleukin-1 beta (IL-1β), interleukin-6 (IL-6) [16], and tumor necrosis factor (TNF) [17] are associated with anxiety behavior and also with more severe depressive disorders. Meanwhile, interleukin-8 (IL-8) levels are related to disorders such as schizophrenia, bipolarity, or autism spectrum disorders [18].

Elevated cortisol levels are observed in disorders such as anxiety and are associated with stress. The higher these levels, the greater the likelihood that an individual will develop depression, as cortisol directly impacts the hypothalamic–pituitary–adrenal (HPA) axis, and when elevated, causes dysregulation of the axis, which promotes the development of mental health issues [19].

Vitamin D increases the expression of genes that encode tyrosine hydroxylase, which is a precursor of dopamine and norepinephrine. Low levels of this vitamin lead to a reduction in these hormones, which in turn manifests depressive symptoms [20].

Dysregulation of the immune response and an increase in the inflammatory cascade can trigger not only elevated cytokine levels but also an increase in C-reactive protein (CRP) and high-sensitivity C-reactive protein (HsCRP) [21]. The induced inflammatory processes cause an increase in nitric oxide production, which activates other cascades and raises cardiovascular risk, detected by troponin, CK-MB, myoglobin, and NT-proBNP, along with the potential presence of a thrombus detected by D-dimer [22].

The use of biomarkers in both basic and clinical research has become so common that their presence as a primary endpoint in clinical trials is almost unanimous [23]. Therefore, it is crucial to find a way to evaluate depression and anxiety quantitatively using a specific biomarker or a panel of biomarkers that can reflect the patients’ state and the effects of therapy [24].

Databases and advanced statistical approaches, such as artificial intelligence (AI) and machine learning (ML), have been successfully implemented in healthcare across various medical fields, such as radiological image analysis, suicide attempt prediction, and forecasting atypical outcomes, among other analyses [25,26]. This data accuracy aims to improve and provide personalized treatments based on each patient’s characteristics, increasing precision and reducing the side effects of generalized treatments [27].

One of the cutting-edge machine learning (ML) methods being applied to predictive models is the Random Forest algorithm, which is a fully non-parametric approach that encompasses different types of responses and outcomes, whether quantitative or qualitative [28,29]. This algorithm is considered to have the best performance by scientists and considers that not all predictor variables are used simultaneously. The recursive partitioning of the dataset is divided into two groups based on a specific criterion until a pre-determined stopping condition is met [30].

Random Forest has the advantage of quantifying the importance of each variable based on the reduction of impurity in the constructed trees. In this way, biomarkers that significantly contribute to the data division are highlighted as the most relevant for the degrees of depression and anxiety. The use of cross-validation ensures that these weights are not the result of random fluctuations, but rather a reflection of consistent relationships in the data [27].

In light of this, this study aims to analyze multiple inflammatory, hormonal, and cardiovascular biomarkers using the Random Forest machine learning algorithm to identify those most relevant in individuals with symptoms of depression and anxiety, assessed through the DASS-21 scale. The analysis also includes a comparison between genders, aiming to identify specific differences in biomarkers, considering that studies have already reported a higher prevalence of depression diagnosis in women compared to men, possibly due to the lower tendency of men to seek help for mental health issues [31].

## 2. Material and Methods

### 2.1. Participants and Eligibility

This study was approved by the Research Ethics Committee of the Federal University of São Carlos (UFSCar) (process number: 54381621.0.0000.5504) and is submitted to the Plataforma Brasil, a national and unified database for research involving human beings, part of the CEP/Conep System. All participants signed informed consent forms and were recruited through convenience sampling as volunteers in psychiatric hospitals and the Psychosocial Care Centers (CAPS) in the interior of the state of São Paulo, Brazil.

Eligible participants for the study were volunteers ages 18 years or older, of both sexes, with a psychiatric diagnosis classified according to the 10th edition of the International Classification of Diseases (ICD-10) based on medical records, and who had no associated comorbidities. Individuals with diagnoses of heart diseases, autoimmune diseases, inflammatory conditions, chronic diseases, and diabetes were excluded.

The volunteers completed the Informed Consent Form (ICF) (Supplementary File S1), which included questions related to personal data, physical health, and mental health. Blood samples were collected once for the analysis of the following biomarkers: D-dimer, NT-proBNP, cortisol, vitamin D, Cardiac Panel (myoglobin, troponin, CK-MB), PCR, and HsCRP, as well as inflammatory cytokines such as IL-6, IL-1β, TNF-α, and IL-8.

A total of 112 volunteers were diagnosed according to the ICD-10 and were assessed for eligibility in 2023. Sixteen were deemed ineligible for the study due to missing information or failure to answer the questions assigned to the study. Therefore, the final sample consisted of 96 volunteers.

### 2.2. Procedures and Instruments

The application of the form and blood collection were carried out on-site at the psychiatric institutions and CAPS, where a mobile laboratory was set up. The data collected included the following constructs: anthropometric characteristics, depression score, anxiety score, alcohol consumption, and smoking. All instruments used in this study were translated and validated for the Brazilian population. Blood samples were also collected from each volunteer for the performance of 13 biomarker tests (described in Section 2.1).

### 2.3. Characteristics of the Volunteers Studied

To characterize the sample, the following data were collected: presence of a psychiatric diagnosis with medical record documentation according to the ICD-10, age (years), sex, weight (kg), height (cm), body mass index (BMI: kg/m^2^), and clinical follow-up with a psychologist and/or psychiatrist.

### 2.4. Depression and Anxiety Score

An adapted version of the Depression, Anxiety, and Stress Scale (DASS-21) was used, consisting of 21 items, with 7 items for each condition. This instrument follows the tripartite model for the clinical assessment of depression, anxiety, and stress, comprising two specific factors (depression and anxiety) and one overlapping (mixed) factor, referred to as the “stressor factor”. The latter is distinct from the others but related due to the conceptual proximity between depression, anxiety, and stress. In Brazil, Vignola and Tucci applied the DASS-21 to adult individuals to investigate the validity and reliability measures of this instrument. The authors confirmed the adequacy of the three-factor model for the samples.

In this study, only the depression and anxiety subscales were used. All items were scored from 0 to 3, indicating the degree to which the individual experienced each symptom described during the previous week, using a 4-point Likert scale ranging from 0 (does not apply to me) to 3 (applies to me most of the time). The total score was obtained by summing the individual scores and was used to classify depression and anxiety symptoms as mild, moderate, or severe [12]. Depression symptoms were classified as follows: no depression: 0–9 points; mild: 10–13, moderate: 14–20, severe: greater than 21. For anxiety, the scoring was as follows: no anxiety: 0–8; mild: 8–9, moderate: 10–14, severe: greater than 15.

### 2.5. Alcohol and Tobacco Consumption Patterns

Cigarette and alcohol consumption were categorized based on the form responses as follows: smoking status was classified as smoker, non-smoker, and quit smoking more than 5 years ago. Alcohol consumption patterns were categorized as non-drinkers or occasional drinkers (with a frequency of up to once a week) and regular drinkers (more than once a week).

### 2.6. Blood Collection

A venous blood sample was collected from each volunteer once, between 7:00 and 10:00 a.m., without the need for the volunteer to be fasting. The serum was separated by centrifugation at 4000× *g* for 10 min. For the rapid test kits (in vitro), whole blood was used immediately after collection to analyze biomarkers such as D-dimer, NT-proBNP, cortisol, vitamin D, Cardiac Panel (myoglobin, troponin, CK-MB), PCR, and HsCRP. For the cytokines IL-6, IL-1β, TNF, and IL-8, serum from each volunteer was used and analyzed by the chemiluminescence method (IMMULITE 1000).

### 2.7. Analysis of the Results

Statistical analysis was performed using Prisma version 9.0. The Shapiro–Wilk test was applied to assess normality. An unpaired t-test with Welch’s correction was used for variables with group distributions. To analyze biomarker behavior, the Random Forest machine learning algorithm was employed. The evaluation metric used was cross-validation. Data are reported as mean ± standard deviation, median [min-max], frequency (proportion), and mean difference (95% confidence interval [CI]).

#### 2.7.1. Random Forest and Cross-Validation

The Random Forest (RF) algorithm is effective for feature selection, as demonstrated in studies on splice site prediction [32] and text classification [33], where it contributed to performance improvements. Both studies highlight its utility in eliminating irrelevant variables and optimizing predictive models.

The data used in this study were obtained from Excel spreadsheets and loaded into the Google Colab environment using the *pandas* library. The dataset included numerical values corresponding to biomarkers and individual depression and anxiety scores. Outliers were retained, as they may reflect real clinical variations. Moreover, no data imputation was required since there were no missing values.

During preprocessing, normalization or standardization of the data was deliberately avoided to preserve the original distribution and, consequently, the clinical interpretability of the biomarkers. The initial selection of biomarkers was based on a systematic literature review, prioritizing those with stronger empirical support. No class balancing techniques were applied, as the focus of the analysis was to assess the relative importance of the biomarkers within the original dataset, rather than to optimize classification metrics.

For modeling, the Random Forest algorithm was employed with five-fold cross-validation, applied separately to depression and anxiety scores. After training, variable importance scores (*feature importances*) were extracted to quantify the relative contribution of each biomarker. Accuracy was recorded for each fold, and the mean accuracy was used to estimate model performance in this exploratory phase.

Although the primary objective was not to predict new cases, this approach demonstrated the model’s ability to capture consistent patterns in the data, thereby reinforcing the robustness of the biomarker importance rankings.

The cross-validation procedure involved splitting the dataset into five subsets (folds), where, in each iteration, four folds were used for training and one for testing. The cross_val_score function from the *scikit-learn* library was employed with the Random Forest classifier, using accuracy as the default metric, defined as the proportion of correctly classified instances. A detailed flowchart of this methodology is available in Supplementary File S2.

#### 2.7.2. Importance Network Between Biomarkers

Spearman’s correlation test was performed to assess the association between the biomarkers tested in female and male volunteers. In all cases, significance was considered at *p* < 0.05. Biomarker importance networks were generated for male and female participants using a circular layout. Each biomarker is displayed as a node arranged around the circle, and the nodesDepression and Anxiety are placed at the center. Edges (lines) connect each biomarker to either Depression or Anxiety, with color (blue for depression, green for anxiety) and thickness proportional to the biomarker’s importance. The color bars on the right indicate the range of importance values, where higher values correspond to more intense color and thicker lines.

## 3. Results

### Characteristics of the Participants

The final sample consisted of 96 volunteers, including 50 females and 46 males. Table 1 provides a descriptive analysis of the participants’ characteristics. The average age of the female group was 41.61 years, while the average age of the male participants was 38 years. Height and weight were measured, and BMI values indicated that both groups were relatively similar, with a mean of 27.27 for females and 26.67 for males.

However, when stratifying by BMI categories, the female group showed a higher prevalence in the “ideal” range (34%) and “overweight” category (32%). In contrast, the male group had a higher prevalence in the “overweight” category (45.65%).

The prevalence of smoking was higher among women, with 72% reporting as smokers, compared to 54.34% of men. Regarding alcohol consumption, the majority of volunteers in both groups reported either no consumption or consuming alcohol only once a week.

Table 2 presents a stratification of depression and anxiety scores assessed using the DASS-21 scale, completed by both groups (women and men). The stratification was categorized as no depression and/or anxiety, mild, moderate, and severe. Among the stratified data, statistically significant differences were observed in the “mild depression” category between women and men (women: mean 12 ± 0.45 vs. men: mean 10 ± 0, *p* < 0.05).

**Figure 1:**
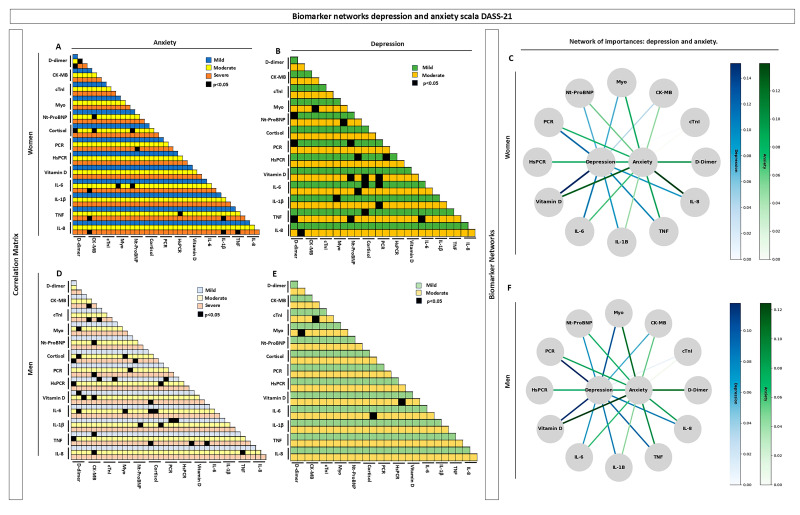
Network of altered interactions among immunological biomarkers in patients with mental disorders. Panels (**A**,**B**,**D**,**E**) illustrate the relevance of immunological biomarkers through a color-coded correlation matrix analysis (left panels), stratified by symptom severity. For women (**A**), levels of anxiety—mild (blue), moderate (yellow), and severe (orange)—are represented in dark shades; for men (**D**), in light shades. Regarding depression, women (**B**) are shown in dark shades for mild (green) and moderate (yellow) levels, while men (**E**) are represented in light shades. Correlations between biomarkers were considered significant when *p* < 0.05 and are highlighted in black. Panels (**C**,**F**) display the biomarker interaction networks built using the Random Forest algorithm. Each node represents a biomarker, including D-dimer, vitamin D, cortisol, NT-proBNP, myoglobin (Myo), CK-MB, troponin I (cTnI), IL-6, IL-1β, TNF, and IL-8. The strength of the connections (green and/or blue) is indicated by the thickness and color of the lines: blue for depression and green for anxiety. The connections for depression and anxiety were assessed on a scale from 0 to 14%, where 0 represents no connection and 14% a strong connection. All analyses were conducted on 96 male and female volunteers who completed an adapted version of the DASS-21 questionnaire to assess symptoms of depression and anxiety.

The analysis of immune biomarker importance, represented in Figure 1, is based on a systems biology approach aimed at exploratory investigation to understand biomarker connectivity among volunteers who completed the DASS-21 questionnaire for depression and anxiety. The graphs in Figure 1 (left panels) were stratified into mild, moderate, and severe levels for anxiety and into mild and moderate levels for depression. These graphs display the correlation of each biomarker’s importance, highlighting statistically significant values (*p* < 0.05) in black for both female and male groups. Figure 1A,D represents the correlations between biomarkers about anxiety. A more pronounced pattern was observed in the male group (Figure 1D) compared to the female group (Figure 1A) across the different levels—mild, moderate, and severe—with emphasis on the biomarkers D-dimer, CK-MB, and cortisol. In the female group, statistically significant results were observed in volunteers with moderate and severe anxiety levels, with emphasis on D-dimer, CK-MB, NT-proBNP, and IL-1β.

The analyses for depression, represented in Figure 1B,E, show through the correlation matrix that the female group presented a higher prevalence of statistically significant values (*p* < 0.05) for both mild and moderate depression levels. Notable biomarkers in this group included NT-proBNP, vitamin D, IL-6, myoglobin (Myo), and CK-MB. In contrast, the male group showed significant results only in individuals with moderate depression, with key correlations observed between Myo and D-dimer, CK-MB and cTnI, cortisol and IL-6, and vitamin D and hsCRP. The biomarker network is represented in Figure 1 (right panel) through nodes, each indicating a specific biomarker analyzed in both female and male groups, without stratification by anxiety or depression severity levels. Instead, individuals were analyzed. Biomarker importance was subcategorized based on percentage and represented by color intensity—blue for depression and green for anxiety.

In the female group (Figure 1C), the highest interaction intensities (%) for depression were observed with IL-6, hsCRP, CRP, vitamin D, and D-dimer. For anxiety, the most relevant biomarkers were IL-8 and vitamin D. In the male group (Figure 1F), interactions for depression occurred among vitamin D, CRP, and D-dimer, while for anxiety, the key biomarkers were vitamin D, myoglobin, and D-dimer.

These data reveal strong connections reflected by higher color intensities for vitamin D in both groups regarding anxiety. For depression, the strongest correlation was observed between vitamin D and CRP, consistently across both groups. These analyses, based on the entire dataset, highlight vitamin D as one of the most relevant biomarkers associated with both anxiety and depression and its shared role across male and female participants.

**Figure 2:**
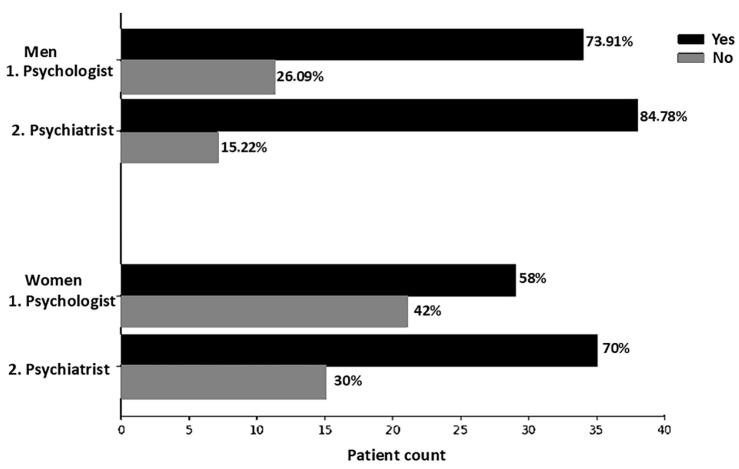
Number of patients who have or have not been seen by a psychologist and/or psychiatrist according to self-report. The figure presents a graph comparing the frequency of men and women who consulted a psychologist and/or psychiatrist. Data are expressed as percentages based on the patient count. Responses are divided into “Yes”, represented by black bars, and “No”, represented by gray bars. The horizontal axis shows the patient count (from 0 to 40), and the vertical axis organizes the categories by gender (men and women), with numbered subdivisions for psychologists (1) and psychiatrists (2).

Figure 2 illustrates the self-reported psychological and/or psychiatric follow-up between the male and female groups when the volunteers are outside the hospital. Among the 46 men assessed, 34 (73.91%) reported undergoing psychological follow-up, and 39 (84.78%) reported psychiatric follow-up. In the female group, 29 women (58%) reported psychological follow-up, and 35 (70%) reported psychiatric follow-up. Overall, the male group demonstrated a higher tendency to seek help compared to the female group.

**Figure 3:**
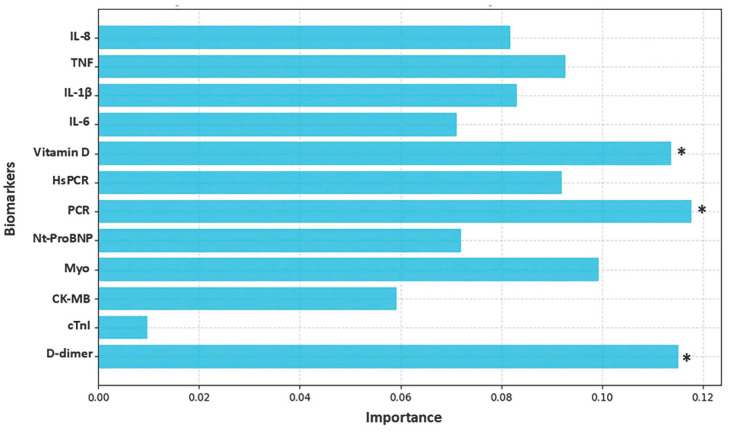
Analysis of biomarker behavior in depression among the male group. The figure shows the relative importance of various biomarkers in the analysis of a predictive model, where the *Y*-axis lists the evaluated biomarkers and the *X*-axis represents the degree of importance assigned to each one. The bars, shown in light blue, indicate the contribution of each biomarker to the model’s performance, highlighting those with greater relevance. The biomarkers analyzed include IL-8, TNF, IL-1β, IL-6, vitamin D, HsCRP (high-sensitivity C-reactive protein), CRP (C-reactive protein), NT-ProBNP (N-terminal pro B-type natriuretic peptide), Myo (myoglobin), CK-MB (creatine kinase MB), cTnI (cardiac troponin I), and D-dimer. The *X*-axis scale ranges approximately from 0.00 to 0.12%, reflecting the magnitude of importance attributed to each biomarker. An asterisk (*) indicates biomarkers that exceeded the 10% importance threshold.

**Figure 4:**
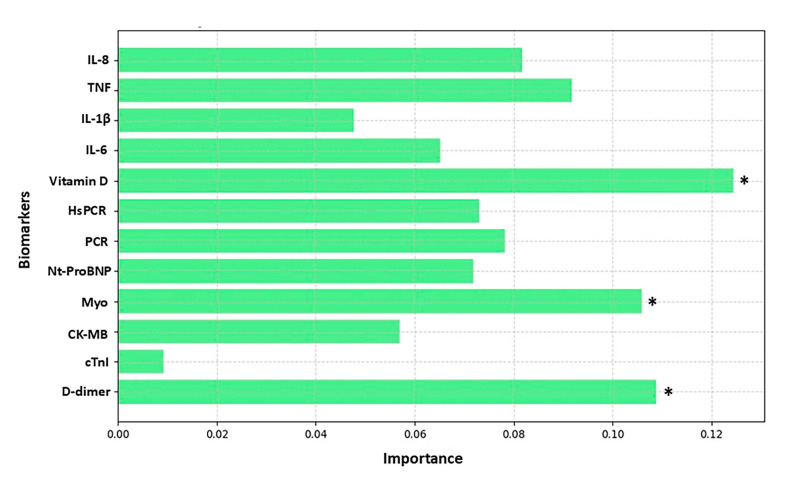
Analysis of biomarker behavior in anxiety among the male group. The figure shows the relative importance of various biomarkers in the analysis of a predictive model, where the *Y*-axis lists the evaluated biomarkers and the *X*-axis represents the degree of importance assigned to each one. The bars, shown in light blue, indicate the contribution of each biomarker to the model’s performance, with emphasis on those with higher relevance. The biomarkers analyzed include IL-8, TNF, IL-1β, IL-6, vitamin D, HsCRP (high-sensitivity C-reactive protein), CRP (C-reactive protein), NT-ProBNP (N-terminal pro B-type natriuretic peptide), Myo (myoglobin), CK-MB (creatine kinase MB), cTnI (cardiac troponin I), and D-dimer. The *X*-axis scale ranges approximately from 0.00 to 0.12%, reflecting the magnitude of importance attributed to each biomarker. An asterisk (*) indicates biomarkers that exceeded the 10% importance threshold.

**Figure 5:**
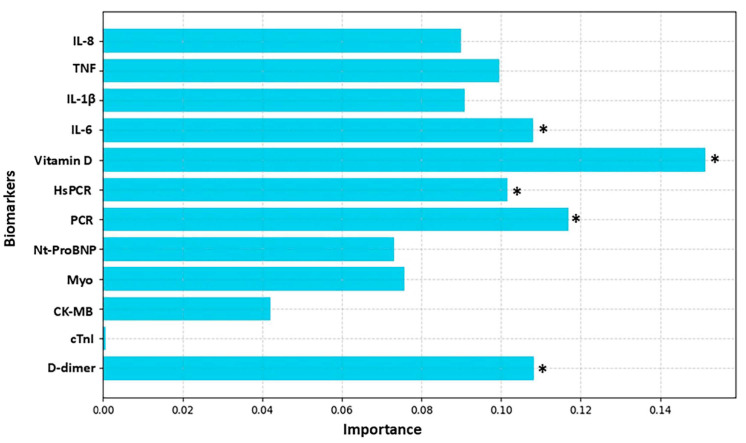
Analysis of biomarker behavior in depression among the female group. The figure shows the relative importance of various biomarkers in the analysis of a predictive model, where the *Y*-axis lists the evaluated biomarkers and the *X*-axis represents the degree of importance assigned to each one. The bars, shown in light blue, indicate the contribution of each biomarker to the model’s performance, highlighting those with greater relevance. The biomarkers analyzed include IL-8, TNF, IL-1β, IL-6, vitamin D, HsCRP (high-sensitivity C-reactive protein), CRP (C-reactive protein), NT-ProBNP (N-terminal pro B-type natriuretic peptide), Myo (myoglobin), CK-MB (creatine kinase MB), cTnI (cardiac troponin I), and D-dimer. The *X*-axis scale ranges approximately from 0.00 to 0.14%, reflecting the magnitude of importance attributed to each biomarker. An asterisk (*) indicates biomarkers that exceeded the 10% importance threshold.

**Figure 6:**
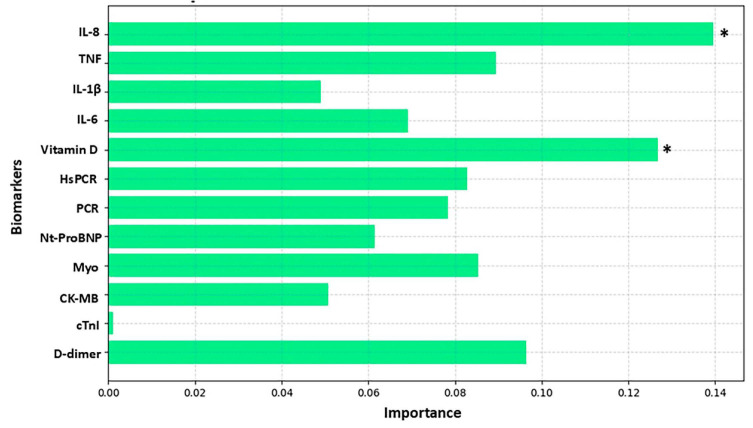
Analysis of biomarker behavior in anxiety among the female group. The figure shows the relative importance of various biomarkers in the analysis of a predictive model, where the *Y*-axis lists the evaluated biomarkers and the *X*-axis represents the degree of importance assigned to each one. The bars, shown in light blue, indicate the contribution of each biomarker to the model’s performance, highlighting those with greater relevance. The biomarkers analyzed include IL-8, TNF, IL-1β, IL-6, vitamin D, HsCRP (high-sensitivity C-reactive protein), CRP (C-reactive protein), NT-ProBNP (N-terminal pro B-type natriuretic peptide), Myo (myoglobin), CK-MB (creatine kinase MB), cTnI (cardiac troponin I), and D-dimer. The *X*-axis scale ranges approximately from 0.00 to 0.14%, reflecting the magnitude of importance attributed to each biomarker. An asterisk (*) indicates biomarkers that exceeded the 10% importance threshold.

Figure 3, Figure 4, Figure 5 and Figure 6 highlight the most relevant biomarkers for depression and anxiety in mental health groups stratified by sex. The analyses were conducted using the Random Forest algorithm, a machine learning approach. Model evaluation was performed using the cross-validation technique, with the average scores serving as a measure of consistency.

Figure 3 illustrates the behavior of the biomarkers most associated with depression in the male group, highlighting vitamin D, PCR, and D-dimer. The cross-validation scores for the depression severity analyzed were [0.7, 0.7, 0.7, 0.7, 0.7]. The average score of 0.7 (or 70%) indicates that the model correctly assigns weights in approximately 70% of the executions on average, meaning each fold was correctly classified in 70% of the cases, demonstrating that Random Forest provides consistent performance in assigning weights for depression severity across different data splits.

Figure 4 highlights the biomarkers associated with anxiety in the male group, with emphasis on vitamin D, myoglobin (Myo), and D-dimer. The cross-validation scores for the severity of anxiety analyzed were [0.6, 0.6, 0.6, 0.5, 0.5], with an average of 0.622 (or 62.22%). This average indicates that the model correctly assigns weights in approximately 62.22% of the executions on average, meaning each fold was correctly classified in 62.22% of the cases. The variation in scores suggests lower stability in assigning weights for anxiety severity compared to depression severity, which may indicate potential areas for model refinement.

Figure 5 illustrates the behavior of the biomarkers most related to depression in the female group, emphasizing IL-6, Hs-CRP, CRP, vitamin D, and D-dimer. The cross-validation scores for depression severity were [0.7, 0.6, 0.4, 0.5, 0.3]. The average score of 0.5 (or 50%) suggests that the model struggles to assign weights consistently. The score variation, ranging from 0.7 to 0.3, may indicate greater variability in the data or challenges in generalizing the assigned weights.

Figure 6 highlights the behavior of the biomarkers most related to anxiety in the female group, with emphasis on IL-8 and vitamin D. The cross-validation scores for anxiety severity were [0.5, 0.5, 0.6, 0.3, 0.6]. The average score of 0.5 (or 50%) indicates that the model has difficulty assigning weights consistently for anxiety data in the female group. The variation in scores, ranging from 0.6 to 0.3, suggests that the model may be facing challenges related to data consistency or the assignment of weights for this category.

## 4. Discussion

Depression and anxiety are highly prevalent health conditions associated with a significant psychosocial burden and a reduction in quality of life. This reduction in quality of life is partly due to the increased vulnerability to the development of other comorbidities, such as obesity, cardiovascular diseases, autoimmune diseases, diabetes, and cancer [34].

Frequently, these conditions present diverse and nonspecific clinical manifestations, making their clinical management quite challenging. Therefore, studies that contribute to the refinement of the diagnosis and monitoring of these diseases are crucial [35].

Currently, the presence of specific biomarkers for depression and anxiety is not well defined. Additionally, the methodology for detecting these analytes, which are typically present in very low concentrations, needs to be improved. Despite these limitations, technological advancements such as machine learning methods are being applied to enhance both the diagnosis and treatment of these disorders [36].

In this study, we assessed which biomarkers are promising in the clinical management of depression and anxiety among men and women with some form of psychiatric diagnosis.

According to Morssinkhof et al. [37], both depression and anxiety are multifactorial conditions influenced by genetics, biological factors such as hormonal fluctuations, and various psychosocial factors [38]. Both conditions are more prevalent in women than in men, and it is believed that this difference arises not only from biological differences but also from the diversity of social roles and the overload of tasks assumed by women in contemporary Western societies [39].

In this context, studies have shown that hormonal fluctuations in women, such as those occurring during the menstrual cycle and menopause, increase stress reactivity, making women more vulnerable to psychiatric disorders like depression. This emotional and physiological hyperarousal affects alertness and attention systems [40]. Moreover, Seeman and González-Rodríguez (2021) highlight that such hormonal variations influence the response to psychiatric treatment, reinforcing the importance of personalized clinical approaches [41].

In this study, the analysis of data by sex revealed that women had a higher prevalence of mild depression compared to men, with no significant differences between the groups for other severities of depressive disorder. Regarding anxiety, women also showed a higher prevalence of moderate severity compared to men. These results align with the literature by showing a higher prevalence of these disorders in women.

Other health conditions amplify the prevalence of depression, especially those associated with inflammation, such as obesity (BMI > 30), autoimmune diseases, and cardiovascular diseases. The incidence of depression and anxiety is heightened by obesity (BMI ≥ 30). The results of this study show that 45.65% of men and 32% of women are overweight, as indicated by their BMI.

High levels of smoking were observed among women (72%), while the rate among men was lower (54%). Nicotine, present in tobacco, is a psychoactive drug with a high potential for addiction. For individuals attempting to quit smoking, lapses or relapses are common, as anxiety symptoms worsen during withdrawal, along with a depressed mood, forming a vicious cycle that increases the need to smoke. The alcohol consumption patterns were also higher in women (80%) compared to men (73.91%), with consumption occurring once a week. However, in cases where alcohol consumption increased to two or more times per week, men had a higher prevalence (26.08%) compared to women (20%).

The treatment for depression and anxiety includes a variety of approaches, both pharmacological and non-pharmacological. Since there is no reliable predictor of treatment response, the choice of therapy can be challenging. In this context, biomarkers, especially inflammatory ones, are of great interest, as they can help patients with a treatment-resistant profile. These biomarkers may provide valuable insights into the underlying biological mechanisms of these disorders, aiding in the development of personalized treatment strategies and improving patient outcomes [37].

Inflammatory blood biomarkers such as IL-6, PCR, and HsCRP are related to depression and anxiety and used to detect multiple diseases. Although they are considered promiscuous markers, combining their results can lead to promising analyses. In other words, there are no specific or agreed-upon biomarkers for depression and anxiety that can be easily tested [37,38].

In recent years, new biomarkers have been identified using ML methods, improving prognosis and treatment for patients [36]. These methods, being more robust, allow for a more subtle and detailed analysis. The Random Forest algorithm, in turn, has been applied in healthcare for various fields, such as oncological applications [39], identification of peptides with binding affinity to target proteins [42], neural networks [43], and support vectors [44], among others.

The Random Forest analyses for depression in this study showed that, when segmenting the results by gender, it was found that although Random Forest was the best model overall, the average scores for the female group were lower than those for men. An analysis of the standard deviation of the biomarkers revealed that, for women, variables such as PCR, HsPCR, vitamin D, IL-6, IL-1B, TNF, and IL-8 exhibited greater variability. This heterogeneity indicates that the data from the female group had greater dispersion, which makes it harder to “stabilize” the model—that is, to achieve cross-validation scores as consistent as those observed for men.

Based on these results, it can be inferred that the clinical manifestation of depression in women is influenced by a greater diversity of factors, and biological aspects may be less impactful compared to men [45]. Roncans et al. (2014) highlight the importance of the social support network as an independent protective factor for depressive disorders in women [46].

For the anxiety analyses, the same difficulty in data attribution for females occurred, with analyses in the male group being more effective.

The onset of depression can be partially explained by the pathophysiological impact of stress and anxiety. Although they are distinct disorders, their etiology involves similar factors, such as genetic predispositions, environmental aspects, and various biological mechanisms, and they are more common in women than in men [47,48].

Some studies describe that vitamin D is related to depression and anxiety by acting on the prefrontal cortex and hypothalamus [49]. Vitamin D induces the expression of tyrosine hydroxylase genes, which are precursors of dopamine and norepinephrine, influencing the serotonergic system that contributes to the maintenance of circadian rhythms. Therefore, altered vitamin D levels may lead to the development of psychiatric disorders, such as anxiety, schizophrenia, alcoholism, and depression [21,50,51].

Immune response has increasingly gained attention in the pathogenesis of psychiatric disorders. The mechanisms can be diverse and involve increased permeability of the blood–brain barrier, dysregulated production of pro-inflammatory cytokines, immune cells, and antibody titers [46]. PCR and HsPCR may be related to the immune-inflammatory response caused by depression and anxiety, as the immune system is imbalanced in the Th1/Th2 response, playing a role in the disease’s pathophysiology [5,52]. The increase in pro-inflammatory cytokines IL-6 and IL-2 affects depression and anxiety in a neuromodulatory way, mediating the neurochemical, neuroendocrine, and behavioral aspects of mental health disorders [53].

Unlike other pro-inflammatory cytokines, IL-8 likely acts over days or weeks, making it potentially specific for more chronic inflammatory alterations in neurodegenerative and neuropsychological diseases in the brain, such as Alzheimer’s disease, schizophrenia, and depression [18].

Regarding the biomarkers D-dimer and Myo, there is limited information on their relationship with the process of depression and/or anxiety. The measurement of D-dimer levels is essential for excluding the diagnosis of venous thromboembolism (VTE) in outpatient, emergency, and hospitalized settings. Individuals with psychosis have a higher risk of VTE, highlighting the importance of screening, even in the absence of symptoms [54].

Parkin L. and colleagues conducted a seven-year study that revealed an increased risk of VTE in individuals using antidepressants, possibly due to the effect of these medications on blood coagulation [55]. In addition to these factors, D-dimer levels can rise with aging, after surgeries, during pregnancy and postpartum, in cases of cancer, chronic inflammatory conditions, and many other disorders [19,49].

Myoglobin, on the other hand, is expressed not only in skeletal muscle cells but also in smooth muscle cells that surround large arteries like the aorta, and it binds to iron to transport and store oxygen [56].

What can be inferred is that the inflammatory process is related to the levels of these biomarkers, as the increase in pro-inflammatory cytokines results in a reduction of nitric oxide, promoting thrombus formation and consequently increasing the risk of cardiovascular events [57].

Despite this challenge, Random Forest remained the best-performing model, demonstrating its ability to handle complex and noisy data. 

## 5. Conclusions

The methodological strategy adopted, based on the use of Random Forest and cross-validation assessment, not only confirmed the robustness of the model but also reliably identified the most important biomarkers for the outcomes studied. Although the greater variability of data in the female subgroup led to lower consistency in the validation scores, the overall results reinforce the validity of the identified patterns, providing a solid foundation for future investigations into the underlying mechanisms of depression and anxiety.

## Figures and Tables

**Table 1:** Descriptive analysis of personal data and lifestyle. Data on age, weight, height, body mass index (BMI), and cigarette and alcohol consumption with results expressed as mean ± standard deviation (SD), median [min–max], frequency (proportion), mean difference (95% confidence interval), and range.

Variables		Woman (N = 50)	Confidence Interval [CI 95%] (Women)	Range (Women)	Man (N = 46)	Confidence Interval [CI 95%] (Men)	Range (Men)
Age (years)		41.61 ± 12.68	[19.00–69.00]	50	38 ± 12.34	[19.00–72.00]	53
Weight (kg)		73.16 ±17.82	[45.00–115.00]	70	78.23 ± 14.83	[54.00–117.00]	63
Height (kg)		163.8 ± 0.066	[152.00–190.00]	0.38	172.82 ± 0.075	[153.00–188.00]	0.35
BMI (kg/m^2^)		27.27 ± 6.507	[17.5–43.82]	26.67	26.16 ± 4.3	[16.41–39.09]	22.68
Obesity degree	Underweight	4 (8%)			2 (4.34%)		
	Ideal	17 (34%)			15 (32.6%)		
	Overweight	16 (32%)			21 (45.65%)		
	Obesity degree I	6 (12%)			6 (13.04%)		
	Obesity degree II	4 (8%)			1 (2.17%)		
	Obesity degree III	3 (6%)			1 (2.17%)		
Smoking	Smoker	36 (72%)			25 (54.34%)		
	Non-smoker	11 (22%)			19 (41.30%)		
	Quit smoking more than 5 years ago	3 (%)			2 (4.34%)		
Alcoholism	Non-drinker or occasional drinker (up to once a week)	40 (80%)			34 (73.91%)		
	Regular drinker (more than once a week)	10 (20%)			12 (26.08%)		

**Table 2:** Depression and anxiety scores according to the DASS-21 questionnaire in both sexes. Data are expressed as mean (M) ± standard deviation (SD), median [min–max], and mean difference (95% confidence interval). Assessment of the questions adapted from the DASS-21 questionnaire. * *p* ≤ 0.05.

Variables	Category	Woman (N = 50)	MD/SD (W)	CI 95% (W)	Man (N = 46)	MD/SD (M)	CI 95% (M)
Depression Score	No depression: 0–9	29 (58%)	2 ± 2.86	0.79 [1.20–2.79]	36 (78.26%)	3 ± 2.64	0.76 [2.23–3.76]
	Mild: 10–13 *	7 (14%)	12 ± 0.45	0.12 [11.87–12.12]	2 (4.34%)	10 ± 0	-
	Moderate: 14–20	14 (28%)	18 ± 2.3	0.63 [7.36–18.63]	7 (15.21%)	17 ± 1.88	0.54 [16.45–17.5]
	Severe: >21	-	-	-	1 (2.17%)	21 ± 0	-
Anxiety Score	No Anxiety: 0–7	28 (60.86%)	2 ± 2.3	0.63 [1.36–2.63]	29 (63.04%)	2 ± 2.4	0.69 [1.30–2.69]
	Mild: 8–9	2 (4.34%)	8.5 ± 0.5	0.13 [8.16–8.83]	5 (10.86%)	8 ± 0.43	0.12 [7.87–8.12]
	Moderate: 10–14	8 (17.39%)	14 ± 1.08	0.29 [13.70–14.29]	8 (17.39%)	11 ± 1.81	0.52 [10.47–11.52]
	Severe: >21	12 (26.08%)	16 ± 2.5	0.69 [15.30–16.69]	4 (8.69%)	16 ± 1.08	0.31 [15.68–16.31]

## Data Availability

The data presented in this study are part of a larger ongoing research project. Due to ethical re-strictions and project agreements, the data cannot be shared publicly at this time. Requests for access to the data may be considered on a case-by-case basis and should be directed to the corresponding author

## Supplementary Materials

The following supporting information can be downloaded at: https://www.mdpi.com/article/10.3390/biom15060793/s1, File S1: The Informed Consent Form; File S2: A detailed flowchart.
