# Supplementary material for: Biomarkers and Mental Disorders: A Relevance Analysis Using a Random Forest Algorithm
Source: Biomolecules. 2025 May 29;15(6):793. doi: 10.3390/biom15060793 (PMC12190397; doi:10.3390/biom15060793)
Supplement: Supplementary file 1 [file biomolecules-15-00793-s001.zip › biomolecules-3632941-File S1.pdf]

DETERMINAÇÃO DE BIOMARCADORES PARA CONFIRMAÇÃO DE DIAGNÓSTICO DE DOENÇAS CARDIOVASCULARES, PULMONARES E SAÚDE MENTAL

* Indica uma pergunta obrigatória

1. E-MAIL

2. CONFIRMAR EMAIL

3. TELEFONE

4. NOME COMPLETO *

5. DATA DE NASCIMENTO *

____

6. SEXO *

Marcar apenas uma oval.

- ( ) Feminino
- ( ) Masculino
- ( ) Outro

7. COR *

Marcar apenas uma oval.

- ( ) Branco
- ( ) Preto
- ( ) Pardo
- ( ) Indígena
- ( ) Outro

8. PESO *

9. ALTURA *

10. PROFISSÃO *

11. FUMA *

Marcar apenas uma oval.

- Não fumo
- Nunca fumei
- Parei de fumar há pelo menos 5 anos
- Parei de fumar durante a pandemia
- Fumo até um maço por dia
- Fumo mais de um maço por dia

12. Padrão de consumo de bebida alcoólica nos últimos 3 meses (considerar uma dose 350ml de cerveja, 150ml de vinho ou 50 ml de destilado) *

Marcar apenas uma oval.

- Não bebo ou bebo menos que 1 vez por mês
- Consumo até 2 vezes na semana, no máximo 3 doses por ocasião.
- Consumo até 2 vezes na semana, 4 doses ou mais por ocasião
- Consumo ao menos 3 vezes por semana, no máximo 3 doses por ocasião
- Consumo ao menos 3 vezes por semana, 4 ou mais doses por ocasião.

13. Padrão de consumo de outras drogas nos últimos 3 meses *

Marcar apenas uma oval.

- Não consumo
- Consumo eventualmente, menos que 1 vez por mês
- Consumo com regularidade, mensalmente
- Consumo com regularidade, entre 1 e 3 vezes na semana
- Consumo com regularidade, ao menos 4 vezes na semana

14. Atividade física (ao menos 30 minutos por ocasião) *

Marcar apenas uma oval.

- Não faço
- Faço 1 ou 2 vezes por semana
- Faço de 3 a 4 vezes por semana
- Faço quase todos os dias da semana

15. Qual atividade pratica? *

____

16. Teve Covid-19? *

Marcar apenas uma oval.

- Sim
- Não
- Talvez
- Mais de uma vez

17. Tomou vacina para Covid-19? / quantas doses *

Marcar apenas uma oval.

- Sim
- Não
- Talvez
- Uma dose
- Duas doses
- Três doses (reforço)
- Quatro doses (reforço)
- Cinco doses
- Não sei

18. Quais vacinas você tomou?

Marque todas que se aplicam.

- [ ] Coronavac
- [ ] Astrazeneca
- [ ] Pfizer
- [ ] Janssen
- [ ] Outra

19. Informações sobre saúde e doenças pré-existentes: Tipo sanguíneo

Marque todas que se aplicam.

- [ ] A
- [ ] B
- [ ] AB
- [ ] O
- [ ] Rh+
- [ ] Rh-
- [ ] Nulo (sangue dourado)
- [ ] não sabe

20. Doenças crônicas pré-existentes *
Assinale todas que correspondam com seu quadro de saúde

Marque todas que se aplicam.

- [ ] Asma
- [ ] Bronquite crônica
- [ ] Enfisema pulmonar
- [ ] Hipertensão pulmonar
- [ ] Doença vascular periférica
- [ ] Doença cerebrovascular (ataque isquêmico transitório, demência vascular)
- [ ] Colesterol alto
- [ ] Diabetes tipo 2 (para Diabetes tipo 1 ver questão sobre doenças autoimunes)
- [ ] Outros tipos de diabetes (gestacional, LADA)
- [ ] Câncer
- [ ] Doença renal crônica
- [ ] Cirrose hepática
- [ ] HIV
- [ ] Anemia falciforme
- [ ] Talassemia
- [ ] Hipotireoidismo
- [ ] Hipertireoidismo
- [ ] Parkinson
- [ ] Alzheimer
- [ ] Osteoporose
- [ ] Nenhuma

21. Caso tenha alguma(s) doença(s) crônica não listada(s) na pergunta anterior, indicá-las

____

22. Com relação às doenças cardiovasculares, assinale as opções que correspondam ao seu quadro de saúde *

Marque todas que se aplicam.

- [ ] AVC
- [ ] Infarto prévio
- [ ] Angina prévio
- [ ] Insuficiência cardíaca
- [ ] Hipertensão arterial
- [ ] Hipertensão arterial resistente (HAR)
- [ ] Nenhuma
- [ ] Outra

23. Sobre doenças pulmonares. *
Tem tosse?

Marcar apenas uma oval.

- ( ) Sim
- ( ) Não

24. Se sim, há quanto tempo? *

____

25. Essa tosse é seca ou apresenta catarro? *

Marcar apenas uma oval.

- ( ) Seca
- ( ) Úmida (com catarro)
- ( ) Não tenho tosse

26. A tosse acompanha febre? *

Marcar apenas uma oval.

- ( ) Sim
- ( ) Não
- ( ) Não tenho tosse

27. Sente falta de ar? *

Marcar apenas uma oval.

- Sim
- Não

28. Se sim, com qual frequência? *

____

29. Doenças autoimunes *
Assinale, se for o caso, as opções de doenças autoimunes que se encaixam com seu quadro de saúde

Marque todas que se aplicam.

- [ ] Lúpus
- [ ] Doença de Crohn
- [ ] Psoríase
- [ ] Doença Celíaca
- [ ] Anemia hemolítica
- [ ] Tireoidite de Hashimoto
- [ ] Artrite reumatoide
- [ ] Vitiligo
- [ ] Diabetes tipo 1
- [ ] Esclerose múltipla
- [ ] Síndrome de jögren
- [ ] Nenhuma

30. Caso tenha alguma(s) doença(s) autoimunes não listada(s) na pergunta anterior, indicá-las

____

31. Assinale as alergias que tem: *
A alergia é uma doença que afeta diretamente o sistema imunológico. Ela consiste em uma reação excessiva a qualquer substância que deveria ser normalmente inofensiva ao organismo.

Marque todas que se aplicam.

- [ ] Respiratórias
- [ ] Alimentares
- [ ] Cutâneas (da pele)
- [ ] Alergia a picada de insetos
- [ ] Alergia a medicamentos
- [ ] Alergia a pelos de animais
- [ ] Nenhuma
- [ ] Outro: ______________________

32. Teve gripe comum nos últimos 6 meses? *

Marcar apenas uma oval.

- ( ) Sim
- ( ) Não
- ( ) Talvez

33. Assinale as doenças endêmicas que já contraiu *

Marque todas que se aplicam.

- [ ] Dengue
- [ ] Esquistossomose
- [ ] Febre maculosa
- [ ] Tuberculose
- [ ] Febre amarela
- [ ] Leishmaniose
- [ ] Malária
- [ ] Chagas
- [ ] Leptospiros
- [ ] Hanseníase
- [ ] Nenhuma
- [ ] Outro: ____________________

34. Assinale dentre as doenças mais prevalentes no Brasil, aquelas que já teve *

Marque todas que se aplicam.

- [ ] Catapora
- [ ] Rubéola
- [ ] Sarampo
- [ ] Caxumba
- [ ] Hepatite
- [ ] Meningite
- [ ] Nenhuma
- [ ] Outro: ____________________

35. Informe os remédios de uso contínuo que toma: *

____

36. Levantamento sobre a sua saúde mental *

Faz acompanhamento Psicológico?

Marcar apenas uma oval.

- ( ) Sim
- ( ) Não

37. Faz acompanhamento Psiquiátrico? *

Marcar apenas uma oval.

- Sim
- Não

38. Por favor, leia cuidadosamente cada uma das afirmações abaixo e escolha o número apropriado 0,1,2 ou 3 que indique o quanto ela se aplicou a você durante a última semana, conforme a indicação a seguir: *
0 - Não se aplicou de maneira alguma
1 - Aplicou-se em algum grau, ou por pouco tempo
2 - Aplicou-se em um grau considerável, ou por uma boa parte do tempo
3 - Aplicou-se muito, ou na maioria do tempo

Achei difícil me acalmar

Marcar apenas uma oval.

- 0
- 1
- 2
- 3

39. Senti minha boca seca *

Marcar apenas uma oval.

- 0
- 1
- 2
- 3

40. Não consegui vivenciar nenhum sentimento positivo *

Marcar apenas uma oval.

- 0
- 1
- 2
- 3

41. Tive dificuldade em respirar em alguns momentos (ex. Respiração ofegante, falta de ar sem ter feito nenhum esforço físico) *

Marcar apenas uma oval.

- ( ) 0
- ( ) 1
- ( ) 2
- ( ) 3

42. Achei difícil ter iniciativa para fazer as coisas *

Marcar apenas uma oval.

- ( ) 0
- ( ) 1
- ( ) 2
- ( ) 3

43. Tive a tendência de reagir de forma exagerada às situações *

Marcar apenas uma oval.

- 0
- 1
- 2
- 3

44. Senti tremores (ex. nas mãos) *

Marcar apenas uma oval.

- 0
- 1
- 2
- 3

45. Senti que estava sempre nervoso (a) *

Marcar apenas uma oval.

- 0
- 1
- 2
- 3

46. Preocupei-me com situações em que eu pudesse entrar em pânico e parecesse ridículo (a) *

Marcar apenas uma oval.

- 0
- 1
- 2
- 3

47. Senti que não tinha nada a desejar *

Marcar apenas uma oval.

- 0
- 1
- 2
- 3

48. Senti-me agitado (a) *

Marcar apenas uma oval.

- 0
- 1
- 2
- 3

49. Achei difícil relaxar *

Marcar apenas uma oval.

- 0
- 1
- 2
- 3

50. Senti-me depressivo (a) e sem ânimo *

Marcar apenas uma oval.

- 0
- 1
- 2
- 3

51. Fui intolerante com as coisas que me impediam de continuar o que eu *
estava fazendo

Marcar apenas uma oval.

- 0
- 1
- 2
- 3

52. Senti que ia entrar em pânico *

Marcar apenas uma oval.

- 0
- 1
- 2
- 3

53. Não consegui me entusiasmar com nada *

Marcar apenas uma oval.

- 0
- 1
- 2
- 3

54. Senti que não tinha nenhum valor *

Marcar apenas uma oval.

- 0
- 1
- 2
- 3

55. Senti que estava um pouco emotivo (a)/sensível demais *

Marcar apenas uma oval.

- 0
- 1
- 2
- 3

56. Sentia que meu coração estava alterado mesmo não tendo feito nenhum esforço físico (ex. Aumento da frequência cardíaca, disritmia cardíaca) *

Marcar apenas uma oval.

- 0
- 1
- 2
- 3

57. Senti medo sem motivo *

Marcar apenas uma oval.

- ( ) 0
- ( ) 1
- ( ) 2
- ( ) 3

58. Senti que a vida não tinha sentido *

Marcar apenas uma oval.

- ( ) 0
- ( ) 1
- ( ) 2
- ( ) 3

59. Agora vamos falar sobre a qualidade do seu sono: *
A pergunta seguinte é relativa aos seus hábitos de sono durante o último mês. Sua resposta deve indicar a lembrança mais exata da maioria dos dias e noites deste período.

Durante o último mês, como você classificaria a qualidade do seu sono de uma maneira geral?

Marcar apenas uma oval.

- Muito boa
- Boa
- Ruim
- Muito ruim

60. As questões a seguir, abordam a sua relação com o trabalho: *
Para cada item, responda conforme a frequência a seguir:
frequentemente;
às vezes,
raramente;
nunca
quase nunca.

Com que frequência você tem que fazer suas tarefas de trabalho com muita rapidez?

Marcar apenas uma oval.

- Frequentemente
- Às vezes
- Raramente
- Nunca
- Não se aplica

61. Com que frequência você tem que trabalhar intensamente (isto é, produzir muito em pouco tempo)? *

Marcar apenas uma oval.

- Frequentemente
- Às vezes
- Raramente
- Nunca
- Não se aplica

62. Seu trabalho exige demais de você? *

Marcar apenas uma oval.

- Frequentemente
- Às vezes
- Raramente
- Nunca
- Não se aplica

63. Você tem tempo suficiente para cumprir todas as tarefas de seu trabalho? *

Marcar apenas uma oval.

- Frequentemente
- Às vezes
- Raramente
- Nunca
- Não se aplica

64. O seu trabalho costuma apresentar exigências contraditórias ou discordantes? *

Marcar apenas uma oval.

- Frequentemente
- Às vezes
- Raramente
- Nunca
- Não se aplica

65. Você tem possibilidade de aprender coisas novas em seu trabalho? *

Marcar apenas uma oval.

- Frequentemente
- Às vezes
- Raramente
- Nunca
- Não se aplica

66. Seu trabalho exige muita habilidade ou conhecimentos especializados? *

Marcar apenas uma oval.

- Frequentemente
- Às vezes
- Raramente
- Nunca
- Não se aplica

67. Seu trabalho exige que você tome iniciativas? *

Marcar apenas uma oval.

- Frequentemente
- Às vezes
- Raramente
- Nunca
- Não se aplica

68. No seu trabalho, você tem que repetir muitas vezes as mesmas tarefas? *

Marcar apenas uma oval.

- Frequentemente
- Às vezes
- Raramente
- Nunca
- Não se aplica

69. Você pode escolher COMO fazer o seu trabalho? *

Marcar apenas uma oval.

- Frequentemente
- Às vezes
- Raramente
- Nunca
- Não se aplica

70. Você pode escolher O QUE fazer no seu trabalho? *

Marcar apenas uma oval.

- Frequentemente
- Às vezes
- Raramente
- Nunca
- Não se aplica

71. Para a série a seguir, as opções de resposta são: Concordo totalmente; *
Concordo mais que discordo;
Discordo mais que concordo;
Discordo totalmente

Existe um ambiente calmo e agradável onde trabalho

Marcar apenas uma oval.

- Concordo totalmente
- Concordo mais que discordo
- Discordo mais que concordo
- Discordo totalmente
- Não se aplica

72. No trabalho, nos relacionamos bem uns com os outros *

Marcar apenas uma oval.

- Concordo totalmente
- Concordo mais que discordo
- Discordo mais que concordo
- Discordo totalmente
- Não se aplica

73. Eu posso contar com o apoio dos meus colegas de trabalho *

Marcar apenas uma oval.

- Concordo totalmente
- Concordo mais que discordo
- Discordo mais que concordo
- Discordo totalmente
- Não se aplica

74. Se eu não estiver num bom dia, meus colegas compreendem *

Marcar apenas uma oval.

- Concordo totalmente
- Concordo mais que discordo
- Discordo mais que concordo
- Discordo totalmente
- Não se aplica

75. No trabalho, eu me relaciono bem com meus chefes *

Marcar apenas uma oval.

- Concordo totalmente
- Concordo mais que discordo
- Discordo mais que concordo
- Discordo totalmente
- Não se aplica

76. Eu gosto de trabalhar com meus colegas *

Marcar apenas uma oval.

- Concordo totalmente
- Concordo mais que discordo
- Discordo mais que concordo
- Discordo totalmente
- Não se aplica

77. Abaixo há uma lista de problemas que as pessoas às vezes apresentam em resposta a uma experiência muito estressante. Por favor, leia cuidadosamente cada problema e escolha um número apropriado 0,1,2, 3 ou 4 para indicar o quanto você tem sido incomodado por este problema no último mês, conforme a indicação a seguir: *

0 - De modo nenhum
1 - Um pouco
2 - Moderadamente
3 - Muito
4 - Extremamente

Lembranças indesejáveis, perturbadoras e repetitivas da experiência estressante

Marcar apenas uma oval.

- 0
- 1
- 2
- 3
- 4

78. Sonhos perturbadores e repetitivos com a experiência estressante *

Marcar apenas uma oval.

- 0
- 1
- 2
- 3
- 4

79. Ter reações físicas intensas quando algo lembra você da experiência estressante (por exemplo, coração apertado, dificuldades para respirar, suor excessivo) *

Marcar apenas uma oval.

- 0
- 1
- 2
- 3
- 4

80. Evitar lembranças, pensamentos, ou sentimentos relacionados à experiência estressante *

Marcar apenas uma oval.

- 0
- 1
- 2
- 3
- 4

81. Em relação a dores, quais sente, diariamente: *

____

Este conteúdo não foi criado nem aprovado pelo Google.

Google Formulários

TERMO DE CONSENTIMENTO LIVRE E ESCLARECIDO – TCLE

LABORATÓRIO DE INFLAMAÇÃO E DOENÇAS INFECCIOSAS - LIDI-UFSCar
Departamento de Morfologia e Patologia da Universidade Federal de São Carlos-UFSCar:
Rodovia Washington Luís, km 235 - CEP: 13.565-905 - São Carlos-SP.

COMITÊ DE ÉTICA EM SERES HUMANOS - CEP-UFSCar
Pró-Reitoria de Pesquisa da Universidade Federal de São Carlos (prédio da reitoria do campus São Carlos). Rodovia Washington Luís, km 235 - CEP: 13.565-905 - São Carlos-SP. E-mail: cephumanos@ufscar.br. Telefone (16) 3351-9685.

COMISSÃO NACIONAL DE ÉTICA EM PESQUISA - CONEP
SRTV 701, Via W 5 Norte, lote D - Edifício PO 700, 3º andar – Asa Norte
CEP: 70719-040, Brasília - DF

Você está sendo convidado a participar voluntariamente da pesquisa: " **Determinação de biomarcadores para detecção precoce de desenvolvimento de doença cardiovascular, pulmonar e estresse emocional"**, que será desenvolvida pelo grupo de pesquisa do laboratório de Inflamação e Doenças Infecciosas da UFSCar, sob a coordenação da Profa. Dra Fernanda de Freitas Anibal, com o objetivo de identificar os principais biomarcadores preditores de doenças pulmonares, cardiovasculares, associadas a um indicador de saúde mental para realizar a validação de um painel destes biomarcadores que sejam eficazes para o monitoramento e identificação precoce destas doenças.

Esta pesquisa foi aprovada pelo Comitê de Ética em Pesquisa em Seres Humanos (CEP) da UFSCar que, vinculado à Comissão Nacional de Ética em Pesquisa (CONEP), e seguindo a Resolução n. 466/12, que tem a responsabilidade de garantir e fiscalizar que todas as pesquisas científicas com seres humanos obedeçam às normas éticas do País, e que os participantes de pesquisa tenham todos os seus direitos respeitados.

Ao participar desta pesquisa, você se beneficiará, de forma gratuita, de avaliação e dados laboratoriais referentes às doenças cardíacas, pulmonares ou relacionadas à saúde mental e estresse, que não estão disponíveis no Sistema Único de Saúde - SUS. Ainda, você irá contribuir com a identificação de biomarcadores para auxiliar no diagnóstico diferencial de doenças específicas dentro da cardiologia, pneumologia e da saúde mental. Além disso, o resultado dos testes realizados nesta pesquisa se tornará um importante subsídio técnico ao seu médico de rotina.

Ao concordar em participar dessa pesquisa, você será submetido, inicialmente, a exames laboratoriais com coleta de sangue (4ml) em frasco apropriado contendo ácido etilenodiamino tetra-acético - EDTA. O risco a você é mínimo com eventual dor ou hematoma no local da coleta, que poderá ser evitado mantendo o braço esticado e leve pressão no local da punção venosa. O sangue coletado será encaminhado ao Laboratório de Doenças Infecciosas alocado na UFSCar, e será armazenado em freezer à -80ºC durante o tempo necessário para a realização de análises de identificação dos seguintes biomarcadores: Dimero-D, Painel Cardiaco, NT-ProBNP, PCR, KC-6 e IL6, através de testes de PCR em tempo real que utiliza o DNA isolado das células sanguíneas. Após a utilização do material biológico, este será encaminhado para incineração conforme normas vigentes de órgãos técnicos competentes e respeitando sua confidencialidade e autonomia.

Após ser selecionado, você passará por anamnese. Ao respondê-la, você poderá sentir um pouco de cansaço ou se sensibilizar com a resposta, podendo

interromper o questionário se achar necessário, mediante aviso aos pesquisadores envolvidos, sem qualquer prejuízo para seu atendimento.

Caso seja necessário, haverá assistência imediata a você – sem ônus de qualquer espécie - prestada para atender complicações e danos decorrentes, direta ou indiretamente, da pesquisa. O Projeto visa se responsabilizar por quaisquer danos decorrentes da pesquisa e coloca-se a disposição em prestar assistência imediata e integral, com acompanhamento, tratamento e orientação, pertinente a cada caso mesmo que posterior ao término da pesquisa, além da garantia de indenização.

Vocês poderão ser incluídos em dois grupos de pesquisa, o grupo controle - formado por aqueles que não apresentam sintomas das patologias que serão estudadas neste projeto - ou o grupo experimental, que inclui aqueles com uma das patologias de interesse.

Você tem a liberdade para aceitar participar ou não da pesquisa e de retirar seu consentimento e seu material biológico em qualquer fase da pesquisa, sem penalização ou qualquer tipo de prejuízo diante da unidade de saúde, universidade ou em relação aos pesquisadores do projeto.

A sua participação é voluntária, ou seja, não haverá nenhum ganho financeiro, ou custo já que a coleta de sangue será realizada no próprio local de sua consulta de rotina, não havendo necessidade de deslocamento em dias diferentes.

O material biológico coletado e os dados obtidos ficarão armazenados sob a responsabilidade dos pesquisadores, seguros de identificação, garantindo o sigilo, o respeito à confidencialidade e à recuperação dos dados, para fornecimento de informações do interesse dos participantes voluntários ou para a obtenção de consentimento específico para utilização futura em nova pesquisa. Os dados coletados serão utilizados pelos pesquisadores apenas para fins científicos.

Caso queira, você poderá solicitar em qualquer momento, informações adicionais sobre os resultados obtidos com a utilização do seu material biológico e as orientações quanto às suas implicações, incluindo aconselhamento genético quando aplicável, utilizando os contatos dos pesquisadores disponibilizados neste TCLE.

No dia de sua consulta e coleta de sangue, você deverá seguir os protocolos da COVID-19 como a utilização de máscara, manter 1,5m de distância entre as pessoas e uso do álcool em gel.

Você receberá uma via deste termo, assinada e rubricada em todas as páginas por você e pelo pesquisador, onde constam o telefone e o endereço do pesquisador principal.

Eu,

____,

"Declaro que entendi os objetivos, riscos e benefícios de minha participação na pesquisa e concordo em participar".

gov.br
Documento assinado digitalmente
FERNANDA DE FREITAS ANIBAL
Data: 14/04/2023 09:04:37-0300
Verifique em https://validar.iti.gov.br

______________________________ ______________________________

Assinatura do pesquisador | Assinatura do participante da pesquisa

DATA:

Telefones e Endereços para Contato
Profa. Dra. Fernanda de Freitas Anibal
Fone: 16-3351-9763
e-mail: ffanibal@ufscar
