# Supplementary figures and images for: Biomarkers and Mental Disorders: A Relevance Analysis Using a Random Forest Algorithm
Source: Biomolecules. 2025 May 29;15(6):793. doi: 10.3390/biom15060793 (PMC12190397; doi:10.3390/biom15060793)

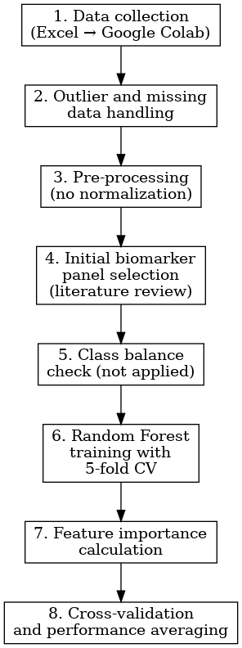

Supplement: Supplementary file 1 [file biomolecules-15-00793-s001.zip › biomolecules-3632941-File S2.tif]
